# Real-Time Optimization of Anti-Reflective Coatings for CIGS Solar Cells

**DOI:** 10.3390/ma13194259

**Published:** 2020-09-24

**Authors:** Grace Rajan, Shankar Karki, Robert W. Collins, Nikolas J. Podraza, Sylvain Marsillac

**Affiliations:** 1 Virginia Institute of Photovoltaic, Old Dominion University, Norfolk, VA 23529, USA; gcher002@odu.edu (G.R.); skarki002@odu.edu (S.K.); 2 Department of Physics and Astronomy, The University of Toledo, Toledo, OH 43614, USA; robert.collins@utoledo.edu (R.W.C.); nikolas.podraza@utoledo.edu (N.J.P.)

**Keywords:** AR coating, ellipsometry, solar cell, CIGS

## Abstract

A new method combining in-situ real-time spectroscopic ellipsometry and optical modeling to optimize the thickness of an anti-reflective (AR) coating for Cu(In,Ga)Se_2_ (CIGS) solar cells is described and applied directly to fabricate devices. The model is based on transfer matrix theory with input from the accurate measurement of complex dielectric function spectra and thickness of each layer in the solar cell by spectroscopic ellipsometry. The AR coating thickness is optimized in real time to optically enhance device performance with varying thickness and properties of the constituent layers. Among the parameters studied, we notably demonstrate how changes in thickness of the CIGS absorber layer, buffer layers, and transparent contact layer of higher performance solar cells affect the optimized AR coating thickness. An increase in the device performance of up to 6% with the optimized AR layer is demonstrated, emphasizing the importance of designing the AR coating based on the properties of the device structure.

## 1. Introduction

In recent years, thin-film solar cells based on Cu(In,Ga)Se_2_ (CIGS) have developed into a new realm of high efficiencies, with several laboratories able to produce record devices over 22% efficiency after major revisions and alterations of the CIGS deposition process [1]. The typical solar cell structure starts with a substrate (such as glass), a metallic layer serving as back electrical contact (molybdenum), a p-type semiconductor (Cu(In,Ga)Se_2_) that will be the main absorber, a n-type semiconductor (CdS) that will act as a buffer layer and heterojunction partner, a combination of transparent layers serving as the top electrical contact (ZnO and AZO) and a metallic grid for current collection (Ni/Al/Ni). As absorber and buffer layer properties are modified with each enhancement, it is also important to continue to develop better light-trapping strategies as well. The power conversion efficiency of the device can be increased by minimizing the overall reflection losses and with an enhanced short-circuit current density (Jsc) by applying an efficient anti-reflective (AR) coating. Various strategies exist to achieve anti-reflection, including porous/patterned features and coatings with graded properties [2]. AR coatings can themselves be homogeneous or heterogenous, and be made of a single layer or multiple layers. Magnesium fluoride (MgF_2_) is the most widely used AR coating material in CIGS solar cells because it forms high-quality films and has a low refractive index, n [3]. However, the material alone is not sufficient, and a careful deposition process, leading to a precise thickness, is paramount to the successful application of the AR coating. The thickness of the AR coating should be chosen such that destructive interference effects occur between the light reflected from the CIGS cell interface and the AR coating surface, allowing reflections at the specific wavelength to be eliminated. This leads to the condition that the AR layer thickness should equal one-quarter of the wavelength within the coating, or d = λ/4 = λ_0_/4n, where λ_0_ is the wavelength of the wave in vacuum [4]. As more research is being implemented to push the limitations in energy conversion of the devices, improvements are made in the device structure and process parameters to optimize the device efficiency, such as with thinner cadmium sulfide (CdS) heterojunction partner layers or aluminum doped zinc oxide (AZO) window layers, or to reduce process cost, such as through thinning the CIGS absorber layer. Various studies have been performed to optically simulate the external quantum efficiency (QE) spectra of high-efficiency CIGS-based solar cells incorporating the effects of variation in the compositional profile of CIGS and thicknesses of various solar cell component layers [5,6,7]. However, for all these alterations, the role and importance of the AR coating are always overlooked. Here, we describe a method based on an optical model and in-situ real-time spectroscopic ellipsometry (RTSE) to accurately model the thickness of the AR coating subject to the effects of the underlying structure of the particular device and implement AR coating deposition to the appropriate thickness for that underlying device structure. In this way, a generalized approach applicable to account for subtle deviations in CIGS device layer thicknesses as well as adaptable to other solar cell architectures is developed. 

## 2. Materials and Methods 

CIGS absorber layers were prepared by a three-stage co-evaporation process in a high vacuum chamber by co-evaporating Cu, In, Ga, and Se on molybdenum (Mo)-coated soda lime glass (SLG). After the CIGS deposition, the samples were dipped into a chemical bath to deposit a thin CdS buffer layer to form the heterojunction. Highly resistive ZnO layers along with more conductive AZO layers were deposited by RF sputtering to obtain a transparent window layer. Ni/Al/Ni grids were e-beam evaporated for the front electrical contacts. MgF_2_ layers were deposited as the anti-reflective coating on the CIGS solar cells by e-beam evaporation and variations in reflectance were assessed for different wavelengths during deposition by RTSE. RTSE measurements were performed in situ during film growth, while other ex-situ spectroscopic ellipsometry (SE) measurements were made post-processing. A rotating compensator, multichannel ellipsometer with a photon energy range from 0.75 to 6.5 eV and an angle of incidence of 65° has been used for all measurements. The data acquisition time is 2 s in this work. An ellipsometer in this configuration measures the full Stokes vector, enabling extraction of ellipsometric spectra and unpolarized reflectance. Quartz crystal microbalances are also used to monitor the deposition rate of the MgF_2_ film. 

Optical models were developed for thin-film multilayer structures for the design and analysis of optical coatings and thin-film solar cells. The structure of the CIGS solar cell, as described above, can be illustrated as a complex multilayer structure. The thickness of the layers including the interface and surface roughness layers as well as the complex index of refraction (*N* = *n* + i*k*) or the complex dielectric constant (*ε* = *ε_1_* + i*ε_2_*) spectra can be extracted by real-time, in-situ or ex-situ SE methods. Based on the deduced optical constants, an optical model is developed to model the CIGS solar cell J_sc_ using transfer matrix theory (TMT). The photovoltaic characteristics were evaluated by external quantum efficiency (QE) measurements (QEX7, PV measurements Inc.) and current density–voltage (J–V) measurements (IV5, PV measurements Inc.) done under simulated AM 1.5G with a light intensity of 100 mW/cm^2^ at 25 °C. The QE measurement system uses a xenon arc lamp source, monochromator, filters and reflective optics to provide stable monochromatic light to the device. A broadband bias light is also used to illuminate the device to simulate illumination conditions similar the J–V measurements. The system uses a detection circuit designed to maximize measurement speed and has a default beam spectral bandwidth of approximately 5 nm. The measurement errors of the two primary metrics considered here for device optimization are typically 0.3% for each QE spectral point and 0.01 mA/cm^2^ for Jsc.

### 2.1. Spectroscopic Ellipsometry Measurements and Data Analysis

SE is a non-invasive technique that measures the change in polarization state of a light beam upon interaction with and reflection from a sample surface. The incident light beam contains measured values that are expressed as the ellipsometric angles ψ and Δ. These values can be related to the components of the electric fields—both parallel (p-) and perpendicular (s-) to the plane of incidence. The change in polarization state describes the properties of the optical system—in this case, a sample [8]—and the measured values are expressed as the ellipsometric angles ψ and Δ. These values can be related to the ratio of Fresnel reflection coefficients $\tilde{R_{p}}$ and $\tilde{R_{s}}$ for p- and s-polarized light. The complex dielectric function ε and the complex index of refraction *N* are related by:

(1) $$\varepsilon_{1}+i\varepsilon_{2}={\left(n+ik\right)}^{2}$$

These representations of optical properties characterize the response of the material in an electromagnetic field. The real part of the complex dielectric function represents the permittivity component that quantifies the stored energy in the fields and the imaginary part represents the dielectric loss factor. These equations describe the interaction of the electromagnetic wave with the electrons in the material [9].

The analysis of ellipsometric spectra employs the Levenberg–Marquardt multivariate regression algorithm to extract the parameters that characterize the multilayer structure including the thicknesses of the bulk and surface roughness and the complex dielectric spectra as a function of photon energy. It is vital to develop a material database of each component of a multilayer stack structure to be able to predict the performance of the device. The interfaces and the surface roughness can entail complicated structural models that introduce ambiguities, which complicates the analysis. Thus, for the initial analysis, the surface roughness or interface roughness layers were not considered in the basic starting model to minimize the complexity and to improve the accuracy of the analysis.

The simplest model with the least number of fitting parameters was considered and the complex dielectric function spectra of the layers were independently obtained by deposition of the materials on well-characterized native oxide-coated silicon wafer substrates. Surface roughness and interface layers for the multilayer structure were modeled using the Bruggeman effective medium approximation as a mixture of the overlying and underlying materials. The effective thickness of a component in the effective medium layer is the product of the effective medium layer thickness and volume fraction of the material. 

**Figure 1:**
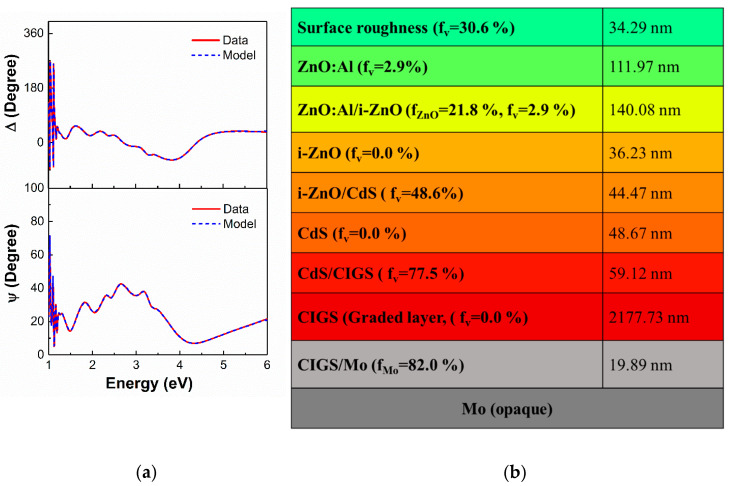
(**a**). Experimental ellipsometric spectra in ψ and ∆ along with the best fit for a specific CIGS solar cell device without an anti-reflective (AR) layer along with (**b**) the layer structure arising from that analysis.

Figure 1 shows the structural model used here for analysis of ellipsometric spectra and results of the analysis for ex-situ SE data of a typical glass/Mo/CIGS/CdS/ZnO/ZnO:Al solar cell film stack. Different parametric models have been developed for materials to extract the complex dielectric spectra as a function of energy for each component material. For the materials of interest here, a general model describing optical response can be represented as:

(2) $$\varepsilon =\varepsilon_{1}+\varepsilon_{2}=\varepsilon_{\infty }+\frac{A_{1}}{E_{1}^{2}-E^{2}}+\frac{A_{1}}{E_{1}^{2}-E^{2}}+Drude\left(E,A_{2},\Gamma_{2}\right)+L\;\left(E,A_{3},E_{3},\Gamma_{3}\right)+CP\;\left(E,A_{4},E_{4},\Gamma_{4},\phi ,µ\right)$$

where the first term represents a constant additive contribution to the real part of the complex dielectric function spectra *ε*_1_, the second and third terms represent Pole or Sellmeier contributions from optical property features at photon energies greater or lower than the boundaries of the measured spectral range, the fourth term represents a Drude model for free carrier absorption, and the fifth and sixth terms represent a Lorentz oscillator and the sum of oscillators describing the critical point (*CP*) electronic transitions. Here, *A*, *E*, Γ, *ϕ*, and *µ* represent the *CP* amplitude or transition strength, resonance energy or position, broadening, phase, and dimensionality exponent, respectively. 

The complex dielectric functions for a Mo layer are obtained by a parametric model consisting of the constant additive term, $\varepsilon_{\infty }$, a Drude contribution for free carrier absorption expected in a metal, and a Lorentz oscillator to describe bound electronic transitions. For a CIGS layer deposited by a three-stage evaporation process, the complex dielectric functions are obtained by numerical inversion of the SE data which are then parametrized based on $\varepsilon_{\infty }$, four CP oscillators, and a Tauc–Lorentz oscillator describing broadband non-parabolic transitions. An Urbach tail is appended to describe the optical response for photon energies below the lowest CPenergy, the direct band gap. The CP resonance energies are 1.19, 1.42, 2.94, and 3.76 eV and the Tauc–Lorentz oscillator resonance energy is 6.23 eV. The Tauc gap E_g_, was equated to the lowest CP energy of 1.19 eV for the CIGS layer. For CdS layer deposited by chemical bath deposition, the complex dielectric functions are obtained by a parametrized model consisting of $\varepsilon_{\infty }$ and two CP oscillators with CP resonance energies 2.38 and 7.24 eV. For the intrinsic ZnO layer, the value of $\varepsilon_{\infty }$ is set to be at unity and three CP oscillators are used along with a Tauc–Lorentz oscillator. The Tauc gap, E_g_, is again equal to the lowest CP energy to avoid the absorption below the direct band gap energy. A single Tauc–Lorentz oscillator and single CP oscillator with resonance energy 3.09 eV are used along with $\varepsilon_{\infty }$ and a Drude contribution to model the complex dielectric functions of the AZO layer. The detailed analyses for all these materials are given in previous papers [6,7,10,11,12,13]. Experimental ellipsometric spectra in terms of ψ and ∆ are shown in Figure 1 along with the best fit obtained by a least squares regression for data collected from a specific complete CIGS solar cell device. 

### 2.2. Transfer Matrix Theory Modeling 

Optical models based on TMT are capable of predicting the reflectance, transmittance, and absorption depth profile as a function of wavelength for multilayer structures and thus provides an improved understanding of the optical losses and gain in the structure [14,15,16,17] Here, we used TMT to calculate the optical interference and absorption in the CIGS solar cell multilayer stack. In this approach, coherent multiple reflections are considered between the planar and transverse electric field components in each layer and are used to calculate the irradiance and absorption [18].

**Figure 2:**
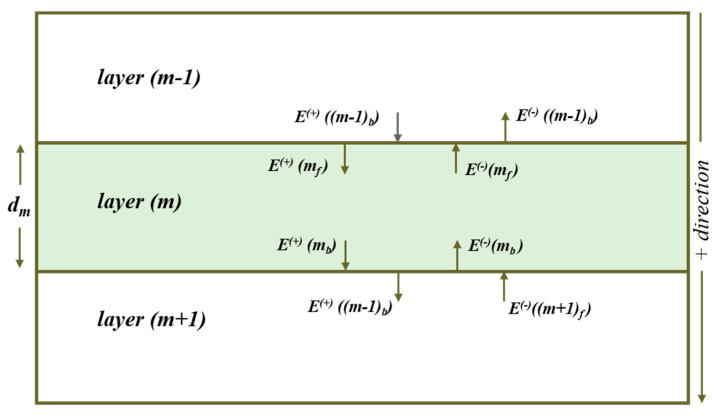
A general multilayer structure having *z* layers of thickness *d_m_* drawn assuming normal incidence.

Consider a light wave normally incident on a multilayer structure composed of *z* layers as illustrated in Figure 2. At each interface, the waves will be propagating in both forward and reverse directions owing to transmission through each layer and multiple reflections at each interface. Maxwell’s equation with appropriate boundary conditions is applied to find the coefficients of reflection and transmission at each interface [15]. These coherent multiple reflections influence optical absorption within the layer and the photogeneration of electron–hole pairs in the solar cell absorber layer specifically. The electromagnetic wave of specular light in the multilayer stack can be described by the amplitudes of the electric field *E*. At any point in the layer *m*, the electric field is represented by four components shown in Figure 2. The electric field magnitude of the light wave propagating in the *m*th layer can be expressed as:

(3) $$E^{\left(+\right)}\left(m_{f}\right)=E^{\left(+\right)}({\left(m-1\right)}_{b}).t_{m-1,m}+E^{\left(-\right)}\left(m_{f}\right).r_{m,m-1}$$

(4) $$E^{\left(-\right)}\left(m_{f}\right)=E^{\left(-\right)}(m_{f}).\tau_{m}$$

(5) $$E^{\left(+\right)}\left(m_{b}\right)=E^{\left(+\right)}(m_{f}).\tau_{m}$$

(6) $$E^{\left(-\right)}\left(m_{b}\right)=E^{\left(-\right)}({\left(m+1\right)}_{f}).t_{m+1,m}+E^{\left(+\right)}\left(m_{b}\right).r_{m,m+1}$$

where *m* corresponds to each layer (ranging from 1, 2… *z*); subscript *f* corresponds to the front (top in Figure 2) and *b* to the bottom of the layer; + and—correspond to the positive and negative direction; *τ_m_* is the phase thickness of each layer; *r_m_* and *t_m_* correspond to the Fresnel’s coefficients. The complex index of refraction of the *m*th layer is defined as *N_m_*. Fresnel’s coefficients relate the amplitude of the reflected and transmitted electric fields to the incident electric field [19].

Under illumination, four electric field amplitudes are associated with each interface for the multilayer structure. Let, $E^{\left(+\right)}\left(m_{f}\right)$ denote the electric field magnitude of light wave that is incident on top of the *m*th interface traveling in a forward direction, $E^{\left(-\right)}\left(m_{b}\right)$ the electric field magnitude of light wave that is incident on the *m*th interface traveling in the backward direction, $E^{\left(+\right)}\left(m_{b}\right)$ the electric field magnitude of light wave that leaves the *m*th interface traveling in a backward direction, and $E^{\left(-\right)}\left(m_{f}\right)$ the electric field magnitude of light wave that leaves the *m*th interface traveling in the forward direction. The matrix representation of electric field magnitude of light wave propagating in the *m*th layer can then be expressed by:

(7) $$\left[\begin{array}{c} E^{\left(+\right)}\left(m_{b}\right)\\ E^{\left(-\right)}\left(m_{b}\right) \end{array}\right]=L_{m+1}\left[\begin{array}{c} E^{\left(+\right)}(\left(m+1{)}_{b}\right)\\ E^{\left(-\right)}(\left(m+1{)}_{b}\right) \end{array}\right]$$

where *L*_*m*+1_ denotes the layer matrix that calculates the amplitudes in the consecutive layers. The layer matrix is the matrix multiplication of the interface matrix (*I*_*m*+1_) and propagation matrix (*P*_*m*+1_). The propagation matrix (*P*_*m*+1_) calculates the electric field amplitudes across the (*m* + 1) layer and the interface matrix (*I*_*m*+1_) calculates the electric field amplitudes across the interface between the *m* and (*m* + 1) layers and can be expressed as follows:

(8) $$P_{m+1}=\left(\begin{array}{cc} e^{-\frac{2\pi N_{m+1}d_{m+1}}{\lambda_{0}}} & 0\\ 0 & e^{\frac{2\pi N_{m+1}d_{m+1}}{\lambda_{0}}} \end{array}\right)$$

(9) $$I_{m+1}=\frac{1}{t_{m,m+1}}\left(\begin{array}{cc} 1 & -r_{m+1,m}\\ r_{m,m+1} & \left(t_{m,m+1}.t_{m+1,m}-r_{m,m+1}.r_{m+1,m}\right) \end{array}\right)$$

(10) $$L_{m+1}=P_{m+1}.I_{m+1}$$

The relationship between the incident, reflected, and transmitted amplitudes is given by [18]:

(11) $$\left[\begin{array}{c} I\\ R \end{array}\right]=\left[\begin{array}{c} E^{\left(+\right)}\left(1_{f}\right)\\ E^{\left(+\right)}\left(1_{b}\right) \end{array}\right]=I_{1}\left[\begin{array}{c} E^{\left(-\right)}\left(1_{f}\right)\\ E^{\left(-\right)}\left(1_{b}\right) \end{array}\right]=I_{1}P_{1}\left[\begin{array}{c} E^{\left(+\right)}\left(2_{f}\right)\\ E^{\left(+\right)}\left(2_{b}\right) \end{array}\right]=I_{1}P_{1}I_{2}\left[\begin{array}{c} E^{\left(-\right)}\left(2_{f}\right)\\ E^{\left(-\right)}\left(2_{b}\right) \end{array}\right]$$

(12) $$\left[\begin{array}{c} I\\ R \end{array}\right]=I_{1}P_{1}P_{2}\ldots I_{z}P_{z}P_{z}\left[\begin{array}{c} E^{\left(-\right)}\left(n_{f}\right)\\ E^{\left(-\right)}\left(n_{b}\right) \end{array}\right]=\left[\begin{array}{c} S_{11}\;S_{12}\\ S_{21}\;S_{22} \end{array}\right]\left[\begin{array}{c} T\\ 0 \end{array}\right]$$

The scattering matrix for the coherent propagation of light for *z* coherent layers, *S_z_*_,_ calculates the electric field amplitudes across the entire multilayer structure [20]. The zero in the column vector is due to the fact that there is no wave incident on the *z*th interface into the substrate traveling in the backwards direction [16,17]. The energy density of the electric field is then calculated using the Poynting’s vector, *S*, given by:

(13) $$S(m_{f})=\left(\frac{1}{2}Y_{0}n_{m}{\left|E^{\left(+\right)}\left(m_{f}\right)\right|}^{2}\right)-\left(\frac{1}{2}Y_{0}n_{m}{\left|E^{\left(-\right)}\left(m_{f}\right)\right|}^{2}\right)+\left(Y_{0}\kappa_{m}.I_{m}\left(E^{\left(-\right)}\left(m_{f}\right).{\left(E^{\left(+\right)}\left(m_{f}\right)\right)}^{*}\right)\right)$$

(14) $$S(m_{B})=\left(\frac{1}{2}Y_{0}n_{m}{\left|E^{\left(+\right)}\left(m_{b}\right)\right|}^{2}\right)-\left(\frac{1}{2}Y_{0}n_{m}{\left|E^{\left(-\right)}\left(m_{b}\right)\right|}^{2}\right)+\left(Y_{0}\kappa_{i}.I_{m}\left(E^{\left(-\right)}\left(m_{b}\right).{\left(E^{\left(+\right)}\left(m_{b}\right)\right)}^{*}\right)\right)$$

where *Y*_0_ is the optical admittance of the free space. The first term of the Poynting’s vector represents the intensity of the waves propagating in the positive direction, the second term is the intensity in the negative direction, and the third term represents the interference component between the two. Absorption in each layer is calculated from the Poynting’s vectors by:

(15) $$A_{m}=\frac{2}{n_{air}.Y_{0}}\left[S\left(m_{f}\right)-S\left(m_{b}\right)\right]$$

The transfer matrix therefore provides absorbance spectra for each layer of the device. The QE spectrum for the CIGS solar device is then optically predicted as a sum of optical absorption within the CIGS active layer components. It is assumed that each above photon absorbed in the CIGS-containing components will photogenerate electron–hole pairs, yielding the maximum QE spectra for that device configuration. *J_sc_* is then calculated from QE assuming that all photogenerated electron–hole pairs are collected and that the incident irradiance spectrum is known using:

(16) $$J_{SC}=\underset{\lambda }{\sum }\Phi_{AM1.5G}\times EQE\left(\lambda \right)\Delta \lambda$$

For the solar cell devices modeled here, the terrestrial solar irradiance air mass 1.5 spectrum (AM 1.5G) at normal incidence is assumed. This approach can be applied to optimization of solar cell AR and component thicknesses for different irradiance spectra (AM 0 or other) and at different angles of incidence, depending upon the in-field operational conditions of the solar cell. This optical model requires precise parameters, namely the complex optical properties and thicknesses of each layer in the multilayer structure which are extracted using SE measurements, such as shown for the data analysis in Figure 1.

## 3. Results and Discussion

There are many ways of modeling an optimal thickness for AR coating on a CIGS solar cell. The typical method is to assume that all layers are at optimal thickness for a typical device and then deposit a MgF_2_ layer to a pre-determined thickness. High-efficiency CIGS solar cells typically have MgF_2_ thickness ranging from 100 to 105 nm [21,22,23]. Here, we propose a method developed with increasing levels of precision as a combination of TMT prediction and real-time optimization via in-situ RTSE measurements. In this way, the appropriate AR coating thickness for any device structure can be determined.

Once the thickness and the optical properties of the component layers of the solar cell were extracted with SE (Figure 1), the maximum QE and J_SC_ were calculated by assuming specular interfaces between layers [24]. Similarly, the reflectance spectra from the structure can be predicted using this model, which helps to optimize the AR coating. The simulation of QE spectra is based on the assumption that all the photogenerated carriers within the active layers are collected without any recombination. Thus, a comparison between the simulated and experimental QE obtained from the measurement of a completed solar cell device can also provide information on the electronic losses, as well as the spectral dependence of losses [25]. Furthermore, the optical model does not take into consideration scattering of light at rough surfaces and interfaces, thus the modeled QE spectra can provide insight into the gain due to light trapping caused by this scattering.

**Figure 3:**
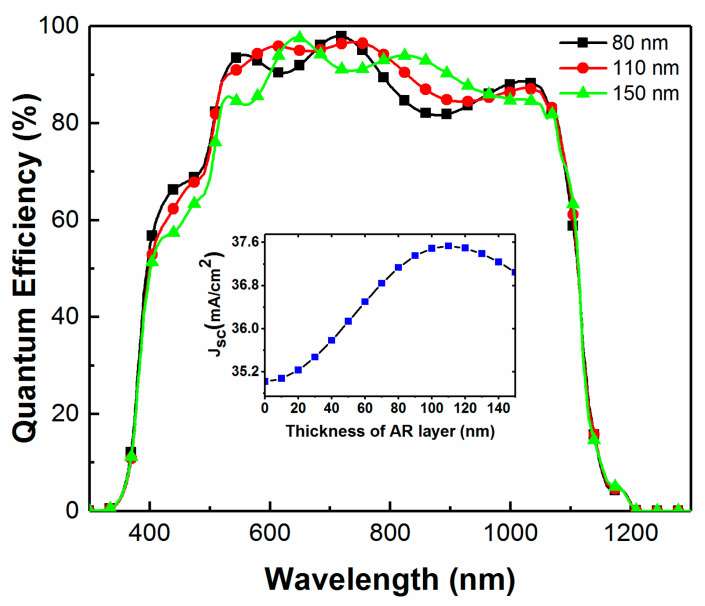
Simulated external quantum efficiency (QE) and short-circuit current density (J_SC_) from TMT models for various thickness of the AR layer for the CIGS solar cell characterized by SE (Figure 1).

This optical model was applied to predict the maximum obtainable J_SC_ for the cell previously analyzed by SE (Figure 1). The variation of the QE and J_SC_ versus the thickness of the AR layer shows that a maximum J_SC_ is predicted for an approximately 110 nm thick MgF_2_ layer for the CIGS device structure (Figure 3).

**Figure 4:**
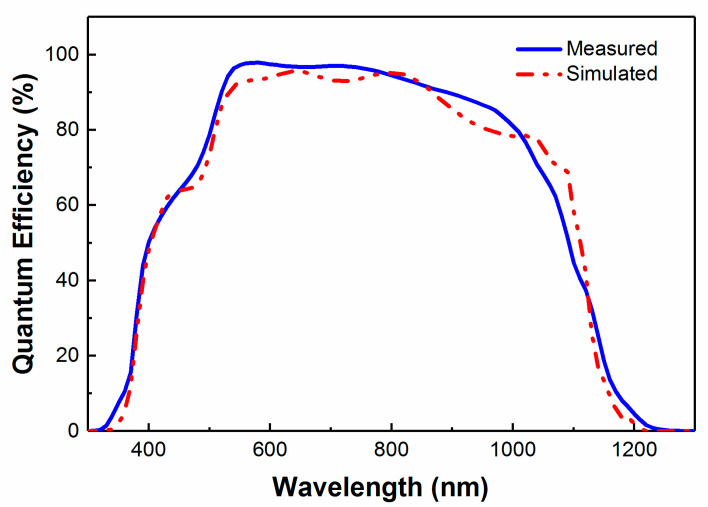
Comparison of the measured and optically simulated QE spectra for the CIGS solar cell characterized by SE in Figure 1.

The model was then verified experimentally by depositing a 110 nm MgF_2_ layer on top of the CIGS solar cell. The calculated and experimentally measured results show good correspondence (Figure 4). The main difference appears in the 500 to 1000 nm spectral range, where the simulated data show pronounced interference fringes due to assumed planar interfaces in the model.

### 3.1. Real-Time Optimization of Thickness of AR Layer via In-Situ RTSE

**Figure 5:**
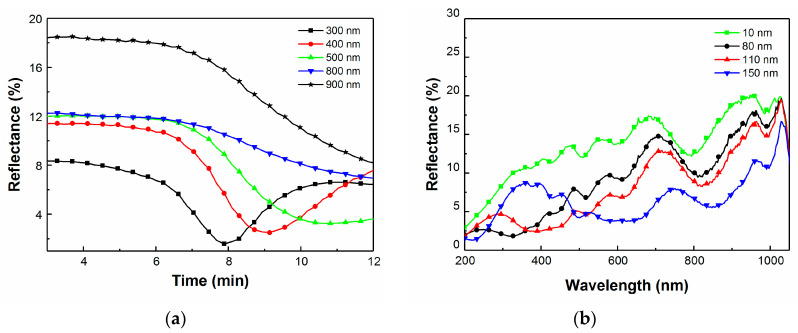
(**a**) Real-time variation of the reflectance during the course of deposition of the AR layer (*t* = 3 to 12 min). (**b**) Real-time variation of the reflectance of the CIGS structure with increased thickness of the AR layer.

Next, we consider the control of the thickness of AR coating during its deposition. A CIGS device without AR coating was loaded in the e-beam evaporation chamber. The reflectance of the device was then monitored in situ and in real time during the deposition of the AR coating on the CIGS device using RTSE (Figure 5). Figure 5a shows real-time measurements of the reflectance from the multilayered CIGS solar cell during the deposition of MgF_2_. Here, reflectance is from the unpolarized irradiance term of the Stokes vector measured at the detector of the ellipsometer divided by the incident irradiance obtained from a calibration using a well-characterized thermal oxide-coated silicon wafer. The irradiance reflected from the calibration sample is divided by the known reflectance of the thermal oxide-coated silicon wafer to deduce the incident irradiance. The variations in reflectance can be observed for different wavelengths during the course of the deposition. A minimum is observed at approximately 8 min for 300 nm, 9 min for 400 nm, and 10 min for 500 nm wavelengths, respectively. In Figure 5b, the reflectance for wavelengths ranging from 300 to 1000 nm is reported for the same device for various thicknesses of the AR coating. It is observed that the average reflectance decreases as the thickness increases up to 110 nm. For larger thicknesses, reflectance increases at low wavelengths and decreases at higher wavelengths. It is therefore difficult to optimize the thickness of the AR coating in real time and in situ without knowledge a priori of which wavelengths are the most crucial to increase the device current (as seen in Figure 3 for example). However, the main advantage of this technique is that it measures reflectance, taking into account scattering at the surface and interfaces, which results in quite different behavior compared to that predicted assuming discrete planar layer boundaries. In Figure 3, the 150 nm AR coating produces alternately higher or lower values of QE compared to that assuming a 110 nm thick AR coating for wavelengths between 500 and 1000 nm, while in Figure 5b the reflectance is systematically lower for an AR coating with thickness of 110 nm.

**Figure 6:**
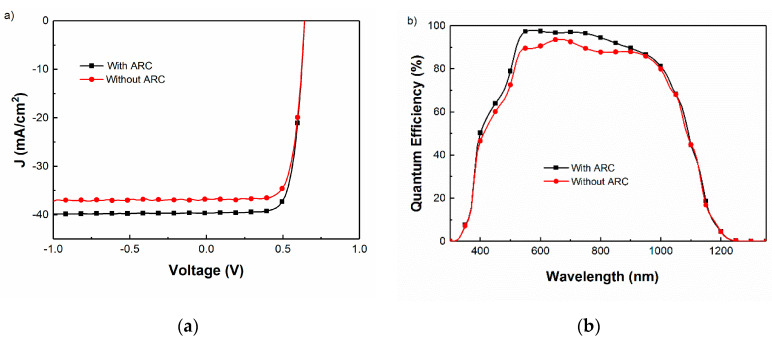
Comparison of measured (**a**) current–voltage (J–V) characteristics under simulated 1-sun illuminations and (**b**) QE spectra obtained for CIGS solar cells with and without the AR coating.

Another sample was overdeposited with a MgF_2_ coating with the focus on minimizing reflectance near the 500 nm wavelength range. The J–V and QE results of the best cell with this optimized coating are shown in Figure 6 and summarized in Table 1, showing enhanced current at all wavelengths as expected, without any substantial change in open circuit voltage (V_oc_) or fill factor (FF).

### 3.2. Real-Time Optimization of Thickness of AR Layer via Transfer Matrix Theory Modeling and In-Situ RTSE for Variation in Multilayer Structure

In this method, both the TMT modeling using SE inputs as well as in-situ RTSE have been used to optimize the thickness of the AR coating based on the underlying layers of the CIGS device. TMT modeling along with SE data allows for accurate prediction of the thickness needed for that particular device, while the in-situ RTSE allows for any experimental issue to be assessed and accounted for in real time. This thickness optimization tool based on optical model and in-situ RTSE is discussed for four different variations: (1) variation in CIGS thickness, (2) variation in CdS thickness, and (3) variation in AZO thickness.

#### 3.2.1. Optimizing the AR Layer as a Function of the CIGS Layer Thickness 

**Figure 7:**
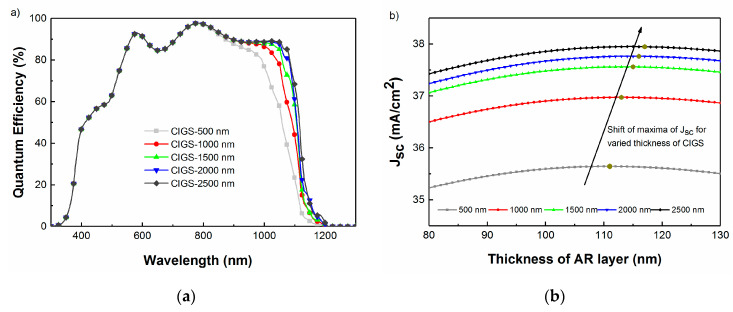
(**a**) Simulated QE with varied CIGS layer thickness and a fixed 111 nm thick MgF_2_ layer. (**b**) Simulated J_SC_ as a function of MgF_2_ thickness, for varied CIGS layer thickness.

A reduction in CIGS layer thickness is of primary interest due to (i) the scarcity of indium, which can have an economic impact on the CIGS solar module production and (ii) as a means to increase manufacturing throughput [26,27]. In this case, only the thickness of the CIGS layer was varied and all the other non-AR coating layers were kept constant. The thickness of AR coating was optimized according to the change in this structure. The J_SC_ and QE of the devices with different CIGS absorber layer thicknesses were modeled using TMT (Figure 7).

The optimum thickness of the MgF_2_ layer is predicted to change from 111 nm for a 0.5 μm CIGS absorber to 117 nm when CIGS layer thickness is increased to 2.5 μm. The loss in J_sc_ occurring as the CIGS thickness is reduced from 2.5 to 0.5 μm is mostly due to incomplete absorption in the CIGS layer, for which a single layer AR coating cannot compensate [28]. It is important to note that this modeling is purely optical and does not take into account potential problems due to back surface recombination and electronic losses, specifically for the ultra-thin solar cell devices.

**Figure 8:**
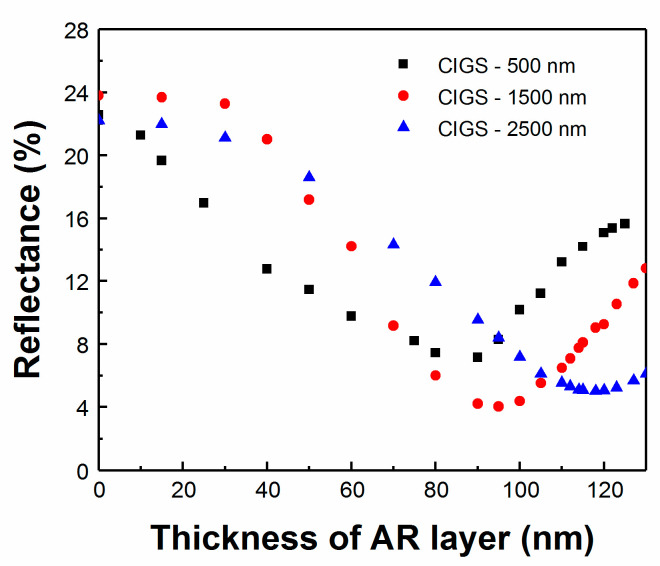
Real-time reflectance for CIGS devices with different CIGS layer thickness.

After the completion of the optical modeling, three devices with 0.5, 1.5, and 2.5 μm thick CIGS absorber layers were fabricated. During e-beam evaporation deposition of the MgF_2_ coating, the reflectance of each device was monitored using RTSE during the deposition of the MgF_2_ AR layer to observe the variation of the reflectance in real time. Spectrally averaged reflectance from the 300 to 1300 nm wavelength range is shown in Figure 8. 

**Figure 9:**
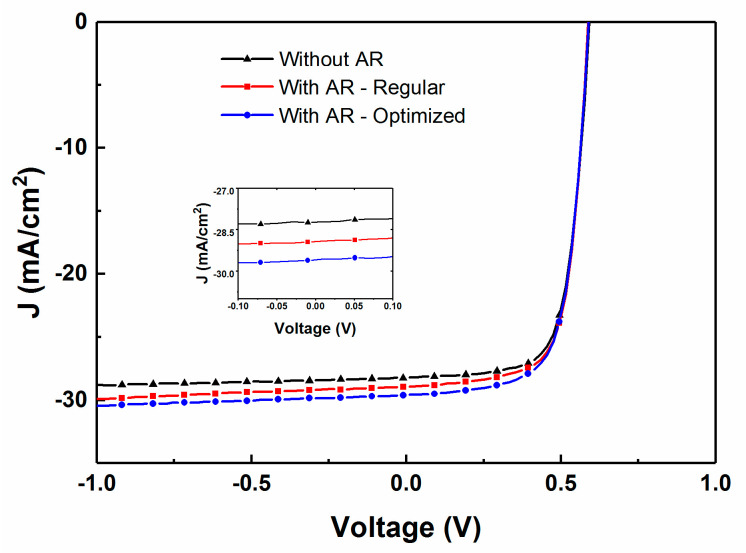
Measured J–V curves for CIGS solar cells with a 1.5 µm thick absorber and either no AR coating, a 105 nm prior modeled AR coating thickness, or a 95 nm in-situ optimized AR coating thickness.

The deposition of the MgF_2_ AR layer was not stopped intentionally at the ideal thickness so as to obtain a clear indication of the optimized thickness. In other runs, this can obviously be modified to stop at the desired ideal thickness, based on the TMT modeling prediction and clear inflection point of the in-situ data. The reflectance minimum does not occur at the same MgF_2_ thickness for all samples with varied thickness of the CIGS layer. In this case, the ideal thickness was extracted to be 88 nm for the 0.5 μm CIGS, 95 nm for the 1.5 μm CIGS, and 117 nm for the 2.5 μm CIGS. Note that this value is slightly different than the one obtained from simulation, probably due to differences in the assumed modeled thicknesses of each layer in the multilayer solar cell structure and the importance of the surface roughness and scattering interfaces, especially for ultra-thin absorbers. The capacity to optimize thickness of AR layer was further tested on CIGS devices with 1.5 μm thick absorbers (Figure 9). Here, several samples from the same deposition were either not coated, coated with a standard 105 nm thick AR, or had a 95 nm thick AR layer determined from optimization of this sample. As shown in the J–V curves, there is an almost 1 mA/cm^2^ increase in J_sc_ for the devices with the optimized AR layer when compared to having the conventional AR layer, with little change observed for the other parameters. 

#### 3.2.2. Optimizing the AR Layer as a Function of the CdS Layer Thickness

**Figure 10:**
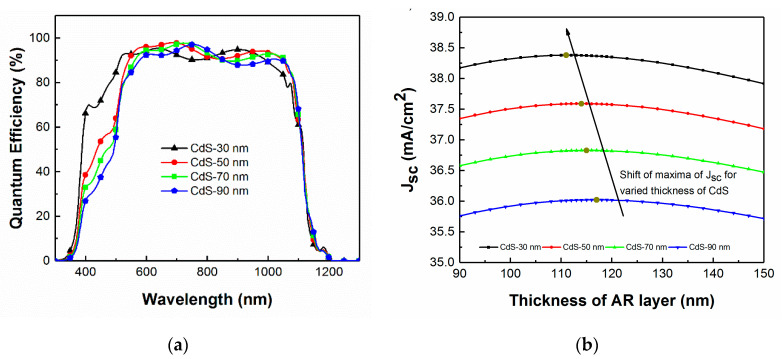
(**a**) Simulated QE with varied CdS layer thickness for a fixed 112 nm thick MgF_2_ layer. (**b**) Simulated J_SC_ as a function of MgF_2_ thickness for various CdS thickness.

Light absorption in the CdS buffer layer is known to cause a significant portion of the total photocurrent loss in the heterojunction device. Photogenerated charge carriers in this buffer layer are only partly collected, reducing the spectral response of the solar cell, notably in the blue region of the solar spectrum at wavelengths less than 500 nm. Figure 10a shows the simulated QE of solar cells with varied CdS layer thickness. The CIGS layer thickness is fixed at 2.5 μm. The blue response of QE decreases with increasing CdS thickness, owing to the increased absorption in the CdS layer. Even though thinning down of the CdS layer seems to be beneficial, there is a lower limit where the interface between CIGS and CdS degrades, upon which electronic losses become substantial. Optical gain and electronic losses must be balanced to fabricate a high-efficiency device. Previous studies suggest that these compensate each other for a CdS layer of thickness of approximately 40 nm [29]. However, with recent key innovation involving alkali post-deposition treatment of the CIGS layer, it is now possible to reduce the minimum thickness of the CdS buffer layer even further to reach higher efficiency [30]. With variation in the CdS layer thickness, it is equally important to optimize the AR coating to ensure the full benefit of these process modifications. Using TMT modeling and SE measurements, the J_SC_ maximum is simulated for various thicknesses of both CdS and MgF_2_ (Figure 10b). The ideal MgF_2_ thickness ranges from 117 nm for 90 nm thick CdS down to 111 nm for 20 nm thick CdS for this specific device.

**Figure 11:**
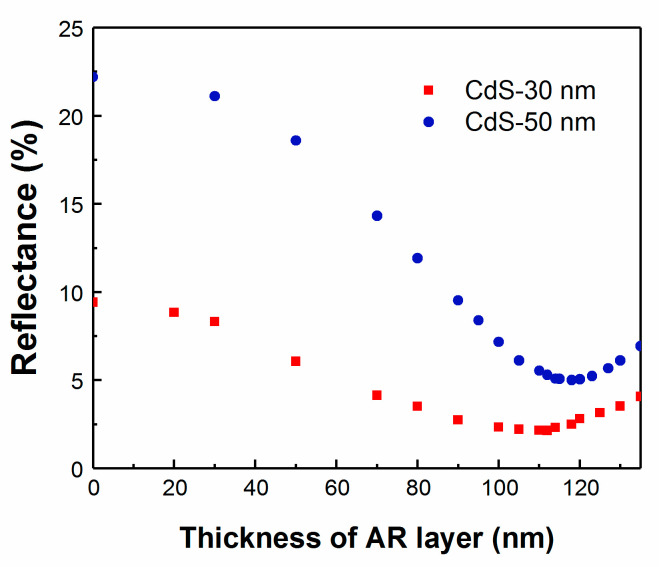
Real-time reflectance for CIGS devices with different layers of CdS thickness.

Following the optical model, two samples were fabricated simultaneously and the thickness of the CdS layer was reduced from 50 nm for a standard device to 30 nm. The reflectance of the device was monitored using RTSE during the deposition of the MgF_2_ AR layer. The deposition of the MgF_2_ AR layer is not terminated at the ideal thickness, to clearly show that a minimum reflectance has been obtained. In Figure 11, the reflectance reached a minimum for the sample with a standard CdS thickness for an AR layer of 118 nm, whereas for the device with reduced CdS thickness, the reflectance reached the minimum for 110 nm. Both thickness values are close to the model prediction. The average reflectance for the device varied with the CdS thickness, but with the deposition of an optimized thickness of AR layer it is possible to reduce the reflectance to obtain the highest efficiency devices in all cases. 

**Figure 12:**
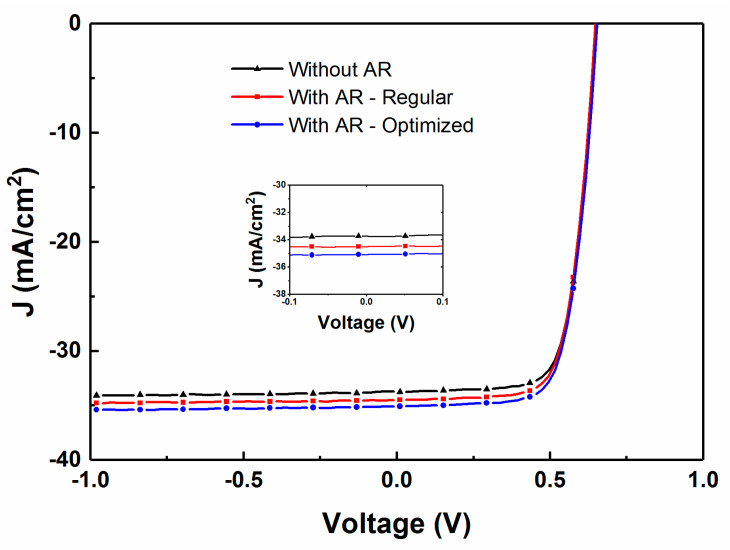
Measured J–V curves for CIGS solar cells with a 30 nm thick CdS layer and either no AR coating, a 118 nm prior modeled AR coating thickness, or a 110 nm in-situ optimized AR coating thickness (insert: close up of the −0.1 V to 0.1 V zone).

The thickness of the AR layer is predicted to be approximately 110 nm for CIGS devices with a thin 30 nm thick CdS layer to maximize the J_SC._ The effect of having deposited the optimized AR layer on the device with thin CdS is demonstrated in Figure 12. There is a clear enhancement in the J_SC_ for the new optimized layer when compared to depositing the standard 118 nm AR layer.

#### 3.2.3. Optimizing the AR Layer as a Function of the AZO Layer Thickness

**Figure 13:**
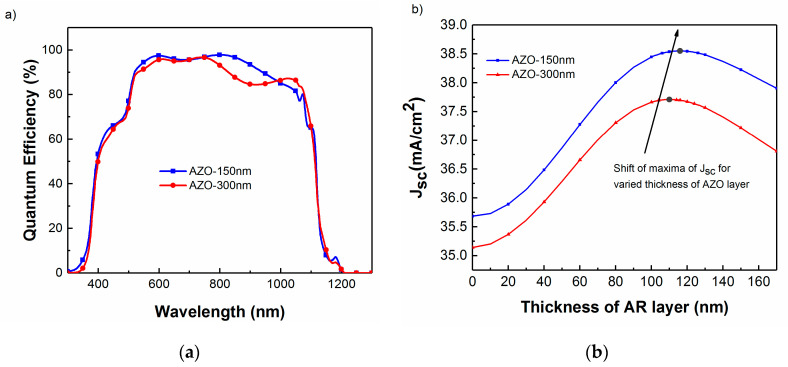
(**a**) Simulated QE for varied AZO layer thickness with a fixed 110 nm MgF_2_ AR layer. (**b**) Simulated J_SC_ as a function of MgF_2_ thickness for various AZO layer thickness.

Similar to the variation in CIGS and CdS layer thicknesses, the AZO layer thickness and optical response is quite often different between one fabrication laboratory and another, without even considering the use of an alternate material such as indium tin oxide. AZO layer thickness is therefore varied and the AR coating is optimized according to the change in the structure. The optical model was used to predict the QE and J_sc_ of the CIGS device with 150 and 300 nm AZO window layer thicknesses (Figure 13). 

As shown in Figure 13, J_SC_ is influenced by the reduction in the thickness of the TCO layer. There is an improved collection efficiency at longer wavelengths for the thinner layer, which can be attributed to the increase in optical transmission by mitigating free carrier absorption to allow more light to be absorbed by the CIGS layer [31]. However, similarly to CdS, one has to take into account the potential electronic losses from reduced current collection. As shown, the ideal MgF_2_ thickness ranges from 110 nm for 300 nm thick AZO up to 115 nm for 150 nm thick AZO for this specific device.

**Figure 14:**
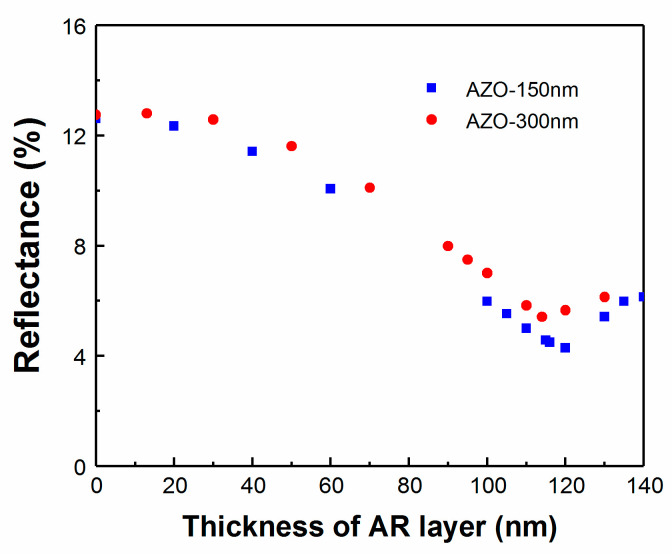
Real-time reflectance for CIGS devices with different AZO layer thickness.

Following this modeling, two samples were fabricated simultaneously and the thickness of the AZO layer was reduced from 300 nm for a standard device to 150 nm. The reflectance of each device was again monitored using RTSE during the deposition of the MgF_2_ AR layer. From Figure 14, the reflectance reached a minimum for the sample with a standard AZO thickness for an AR layer of 110 nm, matching the model prediction. For the device with reduced AZO thickness, the reflectance was the minimum for a 120 nm thick AR layer. The increased AR coating thickness for a thinner AZO layer is also consistent with model prediction. However, with proper deposition of an optimized thickness of AR layer, it is possible to reduce the reflectance to obtain the highest efficiency devices in all cases.

**Figure 15:**
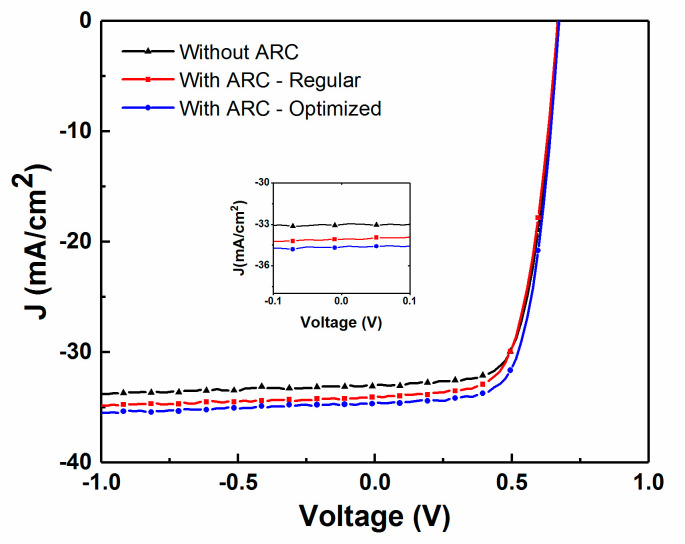
Comparison of the effect of optimized ARC on measured J–V curves for CIGS solar cells with a 150 nm thick AZO layer and either no AR coating, a 105 nm prior modeled AR coating thickness, or a 120 nm in-situ optimized AR coating thickness (insert: close up of the −0.1 V to 0.1 V zone).

Figure 15 compares the effect of optimizing the thickness of the AR layer for a CIGS device with a thinner AZO layer of thickness of 150 nm. The increase in the J_sc_ clearly substantiates the need for depositing a thicker AR layer of 120 nm when compared to the standard thickness of 105 nm as optimized for a regular CIGS device, with a 2–3% increase in J_sc_.

## 4. Conclusions

The critical influence of the thickness of the AR coating for CIGS solar cell application was demonstrated via modeling and experimental results. Using a TMT model, the importance of controlling the deposited MgF_2_ AR layer thickness for improved device performance was demonstrated. The thickness of the AR coating has to be decided based on the underlying structure in the particular CIGS solar cell. The enhancement in the device efficiency with optimized AR layer has been modelled for devices with varied CIGS, CdS, and AZO thicknesses. The experimental results confirmed the validity of the model and allowed for optimized devices to be fabricated. RTSE measured the average reflectance of the CIGS structure to obtain optimum results. In future experiments, these different methods can be integrated to optimize the different layers of various solar cells designs, including those based upon kesterite and organic–inorganic perovskite absorbers.

## Figures and Tables

**Table 1:** Device parameters of the CIGS solar cell before and after depositing the AR coating.

AR Coating	η (%)	J_sc_ (mA/cm^2^)	V_oc_ (V)	FF (%)
Without AR	16.7	35.6	0.64	73.4
With MgF_2_	17.6	37.5	0.64	73.1
